# Assessment and Mitigation of CRISPR‐Cas9‐Induced Nontargeted Translocations

**DOI:** 10.1002/advs.202414415

**Published:** 2025-04-11

**Authors:** Zhiyang Hou, Qiyi Yi, Mengying Wu, Lijun Wu, Fanghua Li, Ting Wang, Po Bian

**Affiliations:** ^1^ School of Basic Medical Sciences Anhui Medical University Hefei 230032 China; ^2^ Hefei Institutes of Physical Science Chinese Academy of Sciences Hefei 230031 China; ^3^ Institute of Physical Science and Information Technology Anhui University Hefei 230601 China; ^4^ Department of Particle Therapy West German Proton Therapy Centre Essen (WPE) West German Cancer Center (WTZ) German Cancer Consortium (DKTK) University Hospital Essen 45147 Essen Germany

**Keywords:** alternative end‐joining, chromosomal rearrangement, CRISPR‐mediated genome editing, DNA single‐strand annealing, nontargeted translocation

## Abstract

The performance of CRISPR‐mediated genome editing near inverted repeats (IRs) potentially results in chromosomal translocations and other catastrophic rearrangements. However, the extent of this risk may be significantly underestimated because current reporter systems focus solely on site‐specific translocations. Here, *trans*‐acting reporter systems in *Escherichia coli* are developed to detect nontargeted translocations. Markedly increased frequency of translocations following CRISPR‐Cas9 activation is observed, with the magnitude determined primarily by the length of the IRs and the proximity between Cas9 target sites and IRs. These translocations arise through a combination of intramolecular single‐strand annealing and alternative end‐joining mechanisms. Furthermore, it is discovered that introducing segments homologous to IR loci can substantially mitigate nontargeted translocations without significantly compromising CRISPR‐Cas9‐mediated editing. The study provides valuable insights into the genetic risks associated with CRISPR technologies and suggests a viable strategy for developing genetically safer CRISPR systems.

## Graphical abstract

Various inverted repeat elements are dispersed throughout genomes. This study reveals that these elements can cause significant chromosomal rearrangements when CRISPR editing occurs in their proximity. The risk can be mitigated by incorporating inverted repeat‐homologous segments into the CRISPR system, which represents a promising strategy for enhancing the genetic safety of CRISPR technologies.

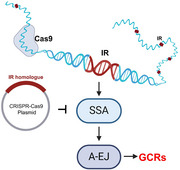

## 1 Introduction

Genome editing using clustered regularly interspaced short palindromic repeats (CRISPR)‐CRISPR‐associated (Cas) proteins has emerged as an indispensable tool in various domains of biomedical research and holds immense potential for revolutionizing the treatment of genetic disorders.^[^ ^1^, ^2^ ^]^ However, there are important concerns regarding its genetic safety. In addition to off‐target effects,^[^ ^3^ ^]^ gross chromosomal rearrangements (GCRs) occurring during CRISPR‐mediated genome editing have become an additional safety‐related aspect.^[^ ^4^, ^5^, ^6^, ^7^, ^8^, ^9^, ^10^, ^11^, ^12^, ^13^ ^]^ These GCRs arise mainly from endogenous DNA repair mechanisms and therefore cannot be easily addressed by modifying the editing tools themselves. CRISPR‐mediated genome editing relies on the repair of Cas9‐cleaved DNA double‐strand breaks (DSBs), but choosing inappropriate repair pathways can lead to genome destabilization.^[^ ^14^, ^15^ ^]^ Several factors can affect this choice, including inverted repeat (IR) motifs in genomes. IRs are prone to adopting non‐B DNA structures, often colocalize with endogenous breakage hotspots, and are associated with chromosomal instability.^[^ ^16^, ^17^, ^18^ ^]^ Notably, they are widely distributed across the genomes of various species, with a frequency of 178 IRs/Mb in humans, 184 IRs/Mb in yeast, and 214 IRs/Mb in *Escherichia coli* (*E. coli*), and are also overrepresented within introns and promoter regions of functional genes.^[^ ^19^, ^20^, ^21^, ^22^ ^]^ Therefore, performing CRISPR‐mediated genome editing near IRs is almost unavoidable.

An increasing body of evidence indicates that the physical proximity between IRs and DSBs has the potential to raise GCRs, thereby highlighting the genetic risk associated with CRISPR‐mediated genome editing.^[^ ^23^, ^24^, ^25^, ^26^, ^27^ ^]^ However, the underlying mechanisms, particularly those governing chromosomal translocations, remain incompletely understood. Translocations represent a significant form of chromosomal rearrangement, wherein one end of one chromosome break incorrectly fuses with a broken end of a different or the same chromosome, often leading to developmental diseases and cancers.^[^ ^28^ ^]^ Nevertheless, current reporter systems are designed primarily to identify site‐specific translocations between two nonhomologous chromosomes and rely on rejoining marker elements/genes located at breakpoints.^[^ ^29^, ^30^, ^31^, ^32^, ^33^, ^34^ ^]^ There is no available reporter system available yet for assessing nontargeted translocations, especially in the wild‐type background.^[^ ^29^, ^35^ ^]^ Consequently, the risk of translocations during CRISPR‐mediated genome editing is significantly underestimated, while potential mechanisms underlying nontargeted translocations might also be overlooked.

The role of IRs in the occurrence of GCRs has primarily been investigated using yeast genetic assays.^[^ ^23^, ^25^, ^26^, ^27^ ^]^ IRs are implicated in two distinct processes: intermolecular single‐strand annealing (SSA) of IRs contributes to the formation of inverted dicentric chromosomes (IDs), and the intramolecular SSA of IRs generates foldback structures (FBs). In wild‐type cells, only mitotic breakages of IDs serve as a source of GCRs, while cleavage of FBs by the Mre11‐Rad50‐Xrs2(MRX)/Sae2 complex results mainly in gene conversion (Figure S1).^[^ ^25^ ^]^ This finding indicates that the intramolecular SSA is rarely responsible for GCRs. In this study, we developed *trans*‐acting reporter systems to enable the detection of nontargeted translocations occurring through a “cut‐and‐paste” mechanism and observed a significant increase in nontargeted translocations when classical CRISPR‐Cas9‐mediated genome editing occurs near IRs. The intramolecular SSA plays a novel role in facilitating nontargeted translocations. Importantly, introducing homologous segments of IR loci into the CRISPR‐Cas9 system can downregulate the intramolecular SSA, mitigating nontargeted translocations.

## 2 Results

### 2.1 Establishment of IR Reporter Systems for Nontargeted Translocations

**Figure 1:**
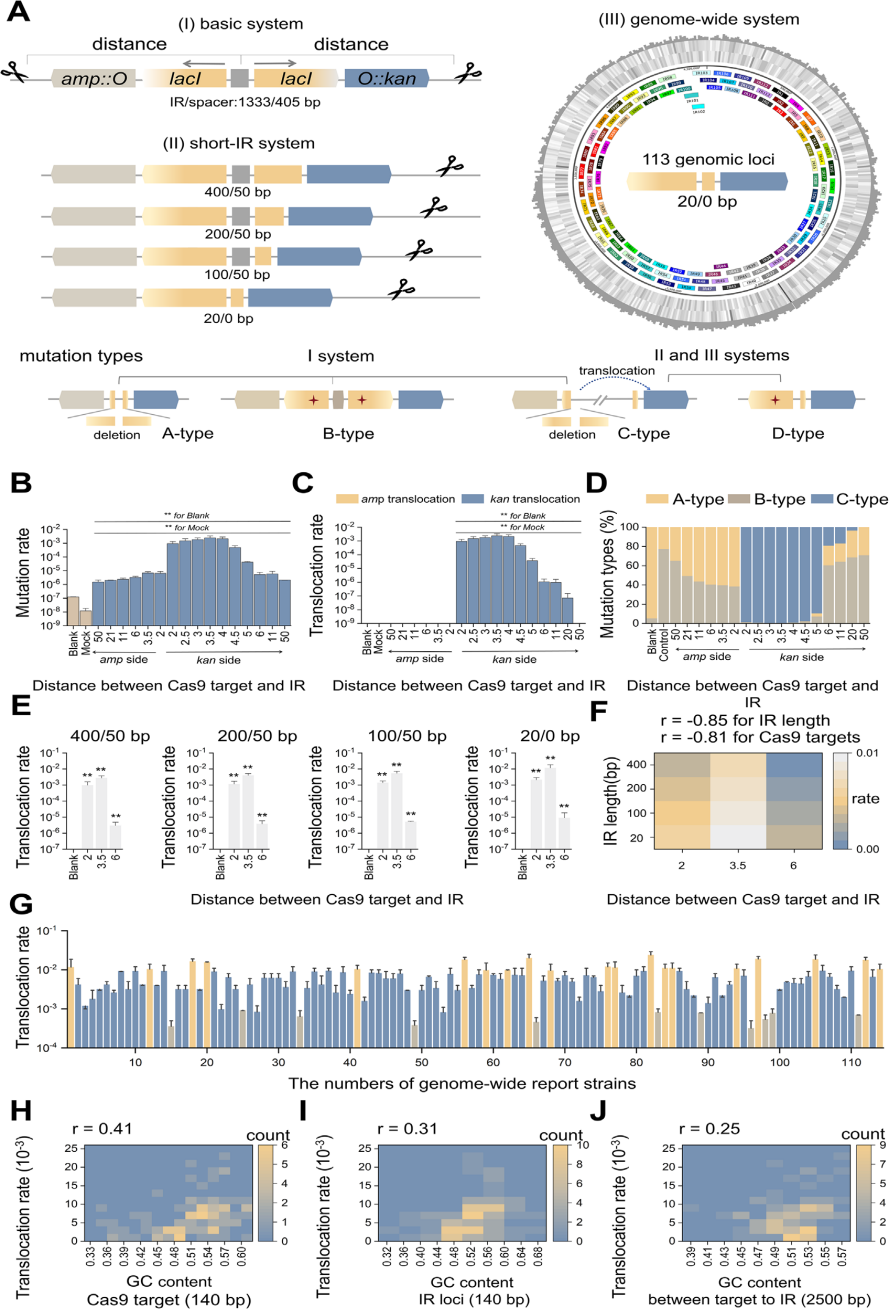
Induction of nontargeted translocations by CRISPR‐Cas9‐mediated genome editing near inverted repeats (IRs). A) Schematic representation of IR reporter systems for nontargeted translocations, including the basic system (I), short‐IR system (II), and genome‐wide system (III). The asterisks in B‐ and D‐types represent point mutations, IR, inverted repeat, *Amp::O, promoter::lacO::amp, and O::kan*, *promoter::lacO::kan*. B) Rates of Amp‐ and Kan‐resistant clones in basic reporter strains subjected to Cas9 cleavage at the indicated distances (*n* = 5, **, *P* < 0.01). C) Translocation rates in the basic reporter strain subjected to Cas9 cleavage (*n* = 5, **, *P* < 0.01). D) Proportions of A‐, B‐, and C‐type mutations in the basic reporter strain (*n* = 5). E) Translocation rates in four short‐IR reporter strains subjected to Cas9 cleavage (*n* = 5, **, *P* < 0.01). F) Heatmap indicating the translocation rates associated with the IR length and distance between Cas9 targets and IRs. G) Translocation rates in genome‐wide reporter strains subjected to Cas9 cleavage at a distance of ≈3 kb (*n* = 5, *P* < 0.01 compared to their respective Blank controls). Heatmaps indicating translocation rates associated with the GC content at Cas9 target sites H) and IR loci I) and between them J). Black, control strains without CRISPR‐Cas9 system. Mock, control strains with CRISPR‐Cas9 system lacking N20 guide sequences.

The bacterial *lac* operon serves as an exemplary model for investigating mutagenesis, comprising the regulator gene *lacI*, the operator *lacO*, and the structural genes *lacZYA*.^[^ ^36^ ^]^ Here, we developed a basic IR reporter system with a dual *lac* operon configuration, where two *lacI* genes were arranged in reverse orientation spaced by a 405‐bp segment, designated *lacI*‐IR; the *lacZ* genes were replaced by ampicillin (Amp) and kanamycin (Kan) resistance genes, respectively (**Figure** **1**A,I). The short‐IR reporter system was subsequently generated on the basis of the basic reporter system by truncating the 3′ end portion of one *lacI* gene, including four reporter strains with different IR lengths (Figure 1A,II). A genome‐wide reporter system was also created by integrating the 20/0‐bp short‐IR region and its flanking *kan* gene into distinct genomic loci, resulting in 113 independent reporter strains (Figure 1A,III). In principle, mutations in both *lacI* genes in the basic reporter system and in the functional *lacI* gene in short‐IR and genome‐wide reporter systems can result in the expression of the *amp* and/or *kan* genes, conferring resistance phenotypes.

A conventional CRISPR‐Cas9/λ‐Red recombineering system was introduced into reporter strains,^[^ ^37^ ^]^ with Cas9 target sites strategically positioned outside the *amp* and *kan* genes. The basic reporter strain exhibited spontaneous mutations characterized by segment deletions spanning two *lacI* genes (A‐type) (Figure 1A; Table S1; Figure S2A, Supporting Information) and double point mutations in the two *lacI* genes (B‐type) (Figure 1A; Table S1; Figure S2B, Supporting Information). Upon activation of the CRISPR‐Cas9 system, additional cut‐and‐paste translocations of the *kan* gene (C‐type) were observed, where the *kan* gene was relocated to other genomic loci, synergistically resulting in structural disruption of both *lacI* genes. In short‐IR and genome‐wide reporter strains, we detected point mutations in the functional *lacI* gene (D‐type) and the aforementioned C‐type mutations (Figure 1A).

### 2.2 Induction of Nontargeted Translocations by Cas9 Cleavage Near IRs

In the basic reporter system, we examined the rate of positive clones exhibiting dual resistance to Amp and Kan following Cas9 cleavage at various distances from the *lacI*‐IR. The rate increased as the distance decreased, but a slight decrease was observed when the distance was less than 3.5 kb (Figure 1B). We specifically analyzed C‐type mutations in these positive clones and found no spontaneous translocations in the absence of Cas9 cleavage. However, translocations were detected when Cas9 cleavage on the *kan* side occurred within a distance of 21 kb. Hereafter, “distance” refers specifically to the distance between IRs and Cas9 targets on the *kan* side. The translocation rates increased as the distance decreased, reaching a maximal value of 2.41×10^−3^ and then saturating within a distance of 4 kb (Figure 1C). When Cas9 cleavage occurred within a distance of 4.5 kb, translocations accounted for more than 98% of the total mutations, but this proportion gradually decreased as the distance increased, reaching zero at 21 kb (Figure 1D). The short‐IR reporter strains also showed no spontaneous translocations; however, Cas9 cleavage at distances of 2, 3.5, and 6 kb induced higher rates than those observed in the basic reporter strain (Figure 1E; Figure S3A,B, Supporting Information). The rate was negatively correlated with IR length (r = ‐0.85) and the distance between Cas9 targets and IRs (r = ‐0.81) (Figure 1F). The essential role of IRs in translocation events was supported by the low translocation rates observed in a non‐IR reporter system (Figure S4, Supporting Information). Additionally, a longer spacer within the IR reduced the occurrence of translocations (Figure S5, Supporting Information).

We further investigated nontargeted translocations initiated at various genomic loci using 113 genome‐wide reporter strains. In these strains, Cas9 cleavage was consistently positioned ≈3 kb away from the IR. Similarly, no spontaneous translocations were detected. Upon activation of the CRISPR‐Cas9 system, varying rates of translocations were observed: an order of magnitude of 10^−2^ in 14% of strains, 10^−3^ in 74%, and 10^−4^ in 12% (Figure 1G; Figure S3C,D, Supporting Information). Furthermore, we evaluated the GC composition at Cas9 targets and IR loci, as well as the intervening regions. The translocation rates exhibited a moderate positive correlation with the GC content within a 140‐bp region centered on Cas9 targets (140 bp, r = 0.41; 560 bp, r = 0.38; 980 bp, r = 0.28) and IR loci (140 bp, r = 0.31; 560 bp, r = 0.27; 980 bp, r = 0.18), but only a weak correlation with the GC content in the intervening regions (r = 0.25), as shown in Figure 1H–J and Figure S3E (Supporting Information). On the basis of these analyses, it can be inferred that factors influencing nontargeted translocations follow this hierarchy: IR length ≥ distance between Cas9 targets and IRs > GC content at Cas9 targets > GC content at IR loci > GC content between Cas9 targets and IRs.

### 2.3 Roles of SSA and Alternative End‐Joining in Mediating Nontargeted Translocations

**Figure 2:**
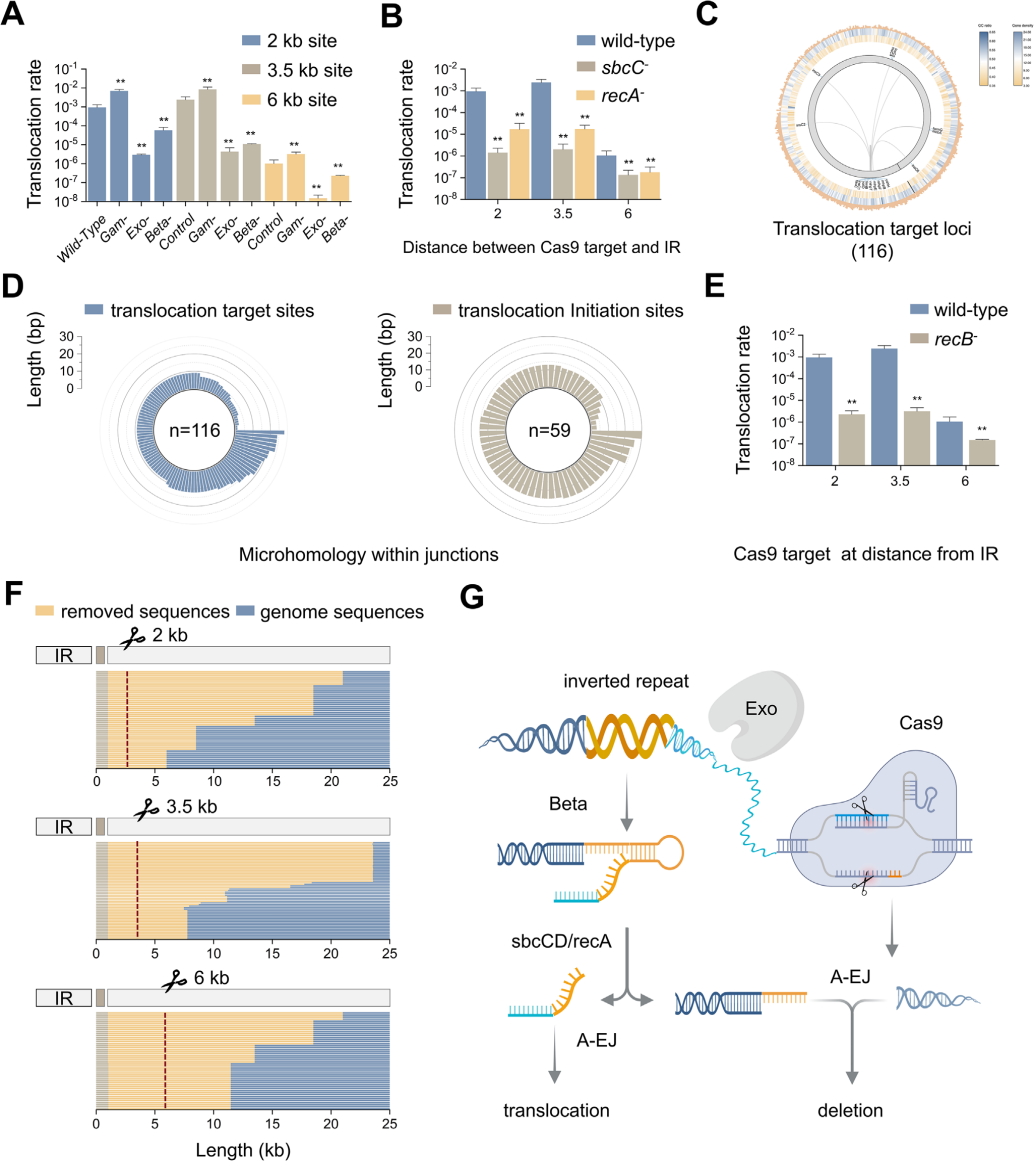
Involvement of single‐strand annealing and alterative end‐joining pathways in nontargeted translocations. Translocation rates of basic reporter strains deficient in λ‐Red recombineering A) and SbcCD and RecA B) (*n* = 5; **, *P* < 0.01). C) Translocation target sites in the genome of *E. coli*. D) Length distribution of homologous sequences within junctions at translocation target and initiation sites. E) Translocation rates in the basic reporter strain deficient in RecB (*n* = 5; **, *P* < 0.01). F) The size of the removed sequences at the translocation initiation sites for Cas9 cleavage at the indicated distances. G) Mechanism underlying nontargeted translocations induced by Cas9 cleavage near the IR.

The CRISPR‐Cas9 system is conventionally employed in conjunction with λ‐Red recombineering (Exo, Beta, and Gam) to increase the efficiency of bacterial DNA recombination.^[^ ^37^ ^]^ Therefore, we examined the role of λ‐Red recombineering in nontargeted translocations and observed a significant decrease in translocation rates in basic reporter strains lacking Exo or Beta (**Figure** **2**A). Given that Exo and Beta are also involved in SSA repair,^[^ ^38^ ^]^ we reasoned that the SSA pathway might be involved in nontargeted translocations. SSA can occur either inter‐ or intramolecularly.^[^ ^25^ ^]^ The intramolecular SSA of IRs typically generates FBs, which can be cleaved by the SbcCD complex and RecA.^[^ ^25^ ^]^ Basic reporter strains deficient in SbcC and RecA presented noticeable reductions in translocation rates (Figure 2B), indicating that intramolecular SSA is crucial for mediating nontargeted translocations.

The next goal was to identify the repair pathways responsible for resolving SbcCD‐ and Cas9‐induced DSBs. We localized the target sites of translocations (Figure 2C) and subsequently analyzed the sequence characteristics of their junctions. Notably, we observed a predominant presence of homologous sequences ranging from 2–28 bp in size, with 93% being less than 20 bp, most of which were less than 9 bp (Figure 2D). The utilization of microhomologous sequences shorter than 20 bp is a characteristic of the alternative end‐joining (A‐EJ) mechanism.^[^ ^39^ ^]^ Furthermore, the basic reporter strain deficient in RecB, an essential upstream component of the A‐EJ pathway, presented reduced translocation rates (Figure 2E). These findings strongly suggest that the A‐EJ pathway might play a crucial role in “pasting” translocated segments into their target sites. Additionally, similar microhomology was observed at translocation initiation sites (Figure 2D), and the “cut” regions extended beyond Cas9 target sites (Figure 2F), indicating the potential involvement of A‐EJ in rejoining IR‐derived and Cas9‐induced DSBs. The entire procedure is schematically illustrated in Figure 2G.

### 2.4 Mitigation of Nontargeted Translocations by Homologous Segments to IR Loci

**Figure 3:**
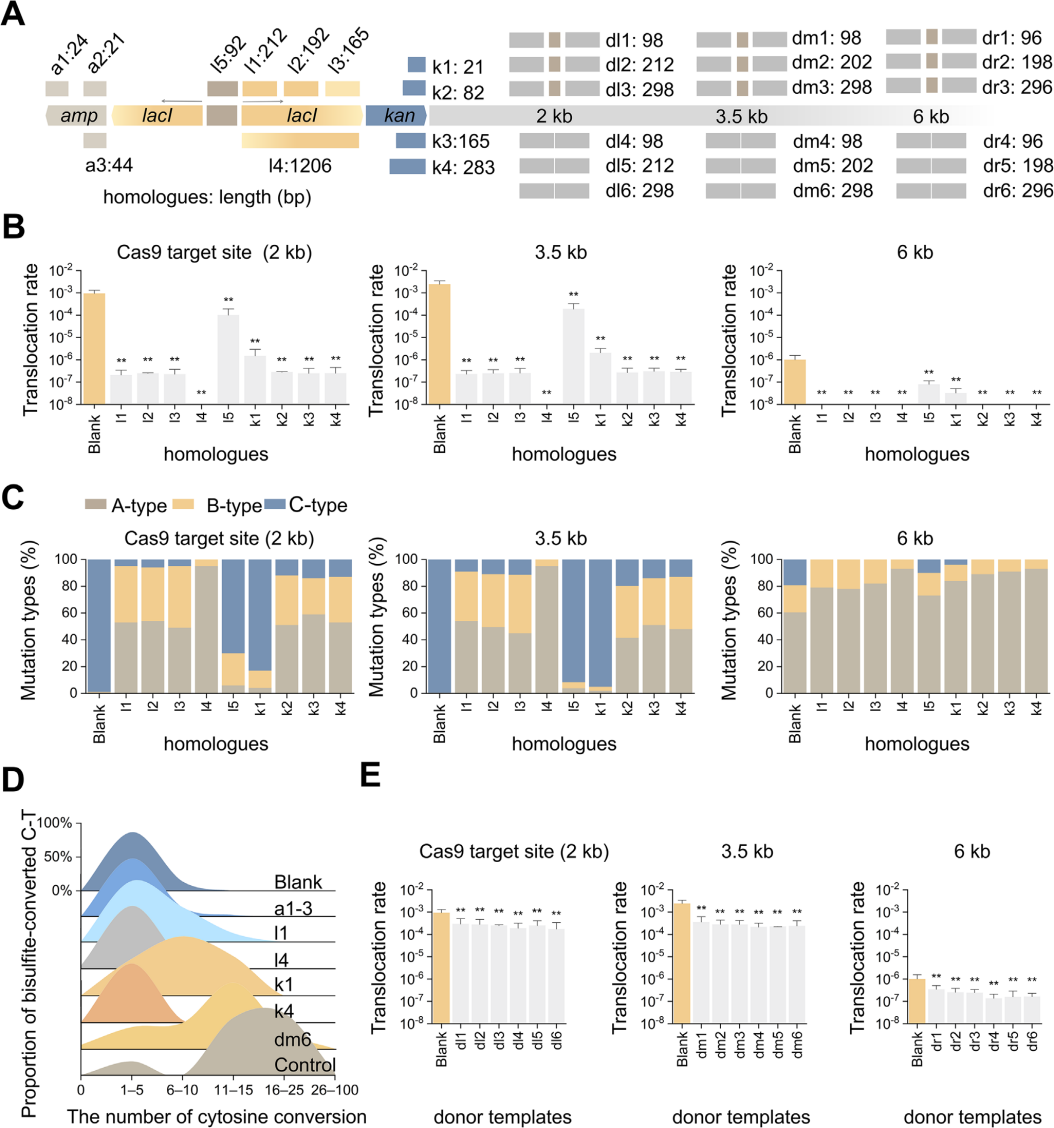
Mitigation of nontargeted translocations by introducing homologues of IR loci. A) Schematic representation of the positions and lengths of *amp* homologues (a1–3), *lacI*‐IR homologues (l1–5), *kan* gene homologues (k1–4), and donor templates (dl1–6, dm1–6, and dr1–6). B) Translocation rates in the basic reporter strain subjected to Cas9 cleavage at the indicted distances from the IR in the presence of various homologues (*n* = 5; **, *P* < 0.01). C) Proportions of three types of mutations in the basic reporter strain subjected to Cas9 cleavage at the indicted distances in the presence of the indicated homologues (*n* = 5). D) The formation of foldback structures, which was dynamically evaluated by the proportional distribution of cytosine conversion in bisulfite‐treated spacers after Cas9 cleavage in the presence of the indicated homologues (*n* = 48). E) Translocation rates in the basic reporter strain subjected to CRISPR‐Cas9‐mediated knock‐in and knockout with various donor templates (*n* = 5; **, *P* < 0.01). Blank, control strains without homologues.

Notably, in the aforementioned experiments, Cas9 cleavage on either the *amp* or *kan* side did not result in any detectable translocations involving the *amp* gene (Figure 1C). In contrast, CRISPR‐Cas9 plasmids contain three short segments (a1–3) completely homologous to the *amp* gene but lack sequences homologous to the *kan* gene (**Figure** **3**A). On the basis of this discrepancy, we reasoned that extrachromosomal segments homologous to IR loci or their flanking regions (hereafter referred to as homologues) could inhibit nontargeted translocations. To test this hypothesis, we introduced homologues of *lacI*‐IR (l1–5) and its flanking *kan* gene (k1–4) into the basic reporter strain along with psgRNA plasmids (Figure 3A). As anticipated, these homologues significantly reduced the occurrence of translocations (Figure 3B,C). The extent of mitigation was influenced by the length and position, with l4, a full‐length *lacI* gene homologue, exhibiting the strongest effect (Figure 3B). Additionally, introducing homologues effectively mitigated translocations in four short‐IR reporter strains and ten randomly selected genome‐wide reporter strains (Figure S6, Supporting Information). These findings suggest that homologues of IR loci can serve as effective inhibitors of nontargeted translocations during CRISPR‐Cas9 genome editing.

In our experiments, homologues were integrated within the nontranscribed region of the psgRNA plasmid, indicating their functional role at the DNA level. Hence, we reasoned that the homologues could inhibit the intramolecular SSA of IRs. To validate this hypothesis, we conducted a structural bisulfite assay to evaluate the impact of homologues a1–3, l1, l4, k1, and k4 on FB formation and observed a significant reduction in FB formation (Figure 3D), which supports our hypothesis.

In precise CRISPR‐Cas9 genome editing, which relies on homology‐directed repair, donor templates are typically designed to be homologous to Cas9 target sites. This raises the question of whether donor templates can effectively mitigate nontargeted translocations. To address this concern, we performed gene knock‐in and knockout experiments at distances of 2, 3.5, and 6 kb from the IR using various donor templates (dl1–6, dm1–6, and dr1–6) (Figure 3A). The results revealed that the presence of donor templates resulted in only a marginal reduction in nontargeted translocations (Figure 3E), likely due to their limited inhibitory effect on FB formation (Figure 3D). These findings suggest that homologues of Cas9 target sites (donor templates) may not possess significant potential for mitigating nontargeted translocations.

### 2.5 No Adverse Effects of Homologues on CRISPR‐Cas9 Genome Editing

**Figure 4:**
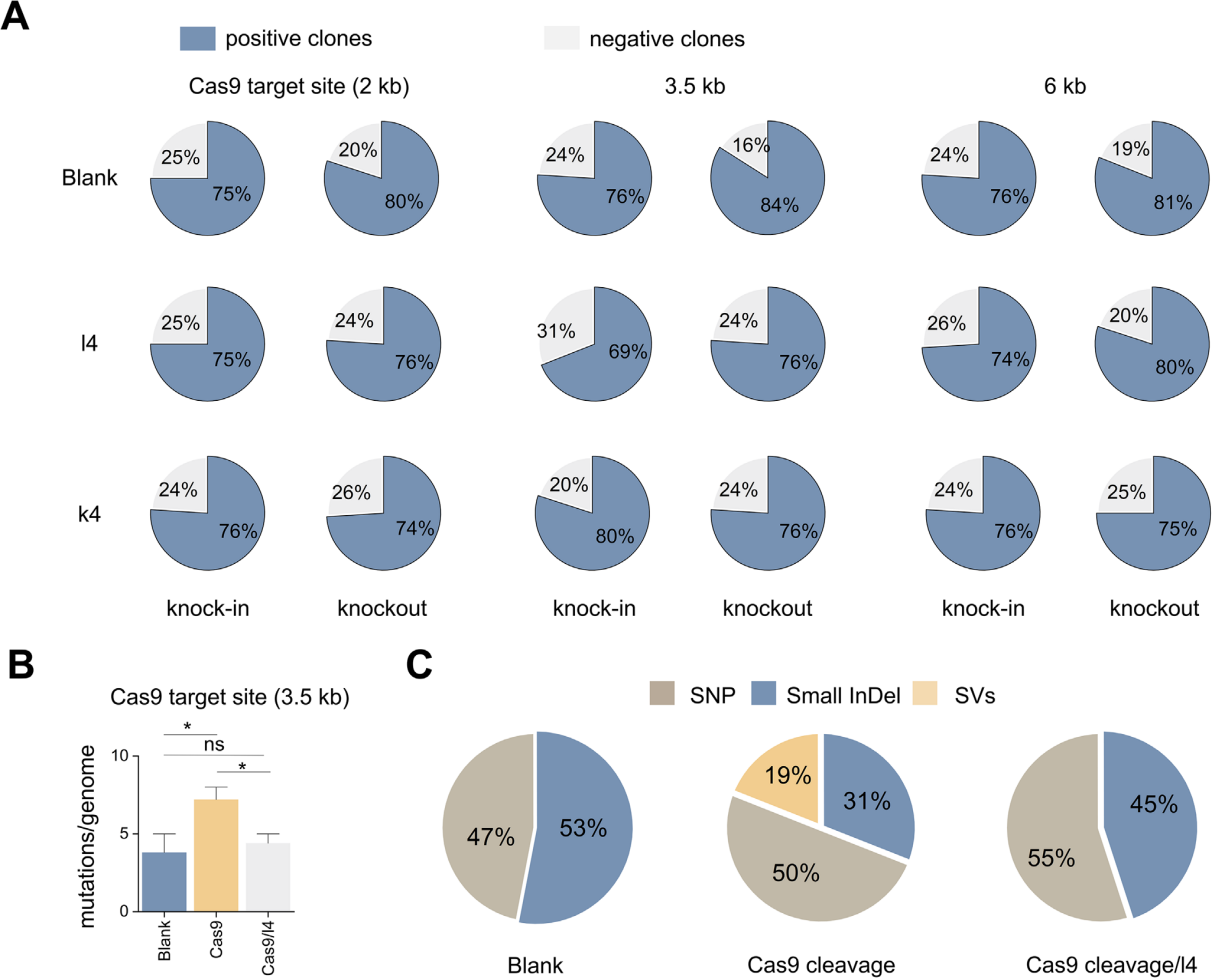
The impacts of homologues on CRISPR‐Cas9 editing outcomes. A) Efficiency of CRISPR‐Cas9‐mediated gene knock‐in and knockout in the presence of homologue l4 (*n* = 144) or k4 (*n* = 144). B) Rates of genome‐wide mutations (*n* = 5, *, *P* < 0.05; ns, *P* > 0.05). C) Proportion of three types of genome‐wide mutations induced by Cas9 cleavage with or without homologue l4 (*n* = 5). Blank, control strains without homologues.

Another pertinent question arises regarding whether the introduction of homologues affects the performance of CRISPR‐Cas9 genome editing. To address this concern, we investigated the impact of homologues on the efficiency and fidelity of CRISPR‐Cas9‐mediated genome editing. Gene knock‐in and knockout experiments were conducted at sites located 2, 3.5, and 6 kb away from the IR using either the CRISPR‐Cas9 system alone or in conjunction with homologue l4 or k4. Our results revealed that the presence of homologue did not significantly alter the efficiency of knock‐in or knockout, as shown in **Figure** **4**A. Additionally, no additional sequence mutations were detected within a 3‐kb region centered on the Cas9 target (3.5 kb) when homologue l4 was introduced, indicating that the inclusion of homologues might not compromise the fidelity of CRISPR‐Cas9‐mediated genome editing.

We also evaluated the potential impact of homologues on genome integrity by conducting whole‐genome sequencing on mutant clones subjected to Cas9 cleavage (3.5 kb) alone or in combination with homologue l4. The application of Cas9 cleavage alone resulted in a significantly higher rate of genome‐wide mutations than did the use of control strains without the CRISPR‐Cas9 system (Figure 4B). These mutations included structural variations (SVs), single‐nucleotide polymorphisms (SNPs), and small insertions/deletions (InDels). The control strains presented only SNPs (47%) and InDels (53%), whereas the strains subjected to Cas9 cleavage alone presented a similar percentage of SNPs (50%) but fewer InDels (31%) along with additional SVs (19%). Notably, the presence of homologue l4 significantly reduced the number of mutation events induced by Cas9 cleavage, preventing the occurrence of SVs in particular (Figure 4C). Compared to control strains, strains subjected to both Cas9 cleavage and l4 showed no significant difference in mutation rate or type. Therefore, introducing homologues of IR loci into CRISPR systems might serve as a viable strategy for mitigating nontargeted translocations.

## 3 Discussion

In this study, we established three types of IR reporter systems to facilitate the detection of nontargeted translocations. Among them, the basic IR reporter system was designed with a dual *lac* operon configuration, specifically incorporating dual resistance genes. In the *lac* operon, mutations in *lacO* can also activate the expression of the *lacZYA* genes. Notably, *lacO* is a hot‐spot for mutagenesis, exhibiting a mutation rate comparable to that of the *lacI* gene.^[^ ^36^, ^40^, ^41^ ^]^ Therefore, if a single *lacO*‐fused resistance gene is used in this reporter system harboring two *lacI* genes, the likelihood of mutations occurring in *lacO* is significantly greater than that of double mutations in both *lacI* genes. To avoid overrepresentation of positive clones resulting from *lacO* mutations rather than from IR mutations, we introduced the dual *lacO‐*fused resistance gene design. This basic reporter system is capable of detecting three distinct types of mutations. Specifically, B‐type mutations exhibit symmetrical point mutation sites and identical mutation forms in the two *lacI* genes, suggesting that these mutations occur through interdependent mechanisms. The proposed mechanism proceeds as follows: an initial point mutation arises in one *lacI* gene and is subsequently “copied” to the corresponding site in the other *lacI* gene via homologous recombination between the two *lacI* genes. As a result, B‐type mutations may predominantly reflect gene conversion events. In principle, C‐type mutations may also involve a special mode in which the left *lacI* gene remains intact during *kan* gene translocations; however, this mode cannot be identified through Amp and Kan resistance selection. To investigate this possibility, we introduced a single‐base frameshift mutation to inactivate the left *lacI* gene in the basic reporter system while preserving the IR structure.^[^ ^42^ ^]^ No intact left *lacI* gene was detected in clones with *kan* gene translocations (Figure S7, Supporting Information), suggesting that structural disruption of IRs might be an essential event in IR‐mediated translocations.

On the basis of these reporter systems, here, we have discovered a novel function of intramolecular SSA in promoting nontargeted translocations. In wild‐type cells, intramolecular SSA‐mediated FBs can be processed into DSBs by SbcCD and RecA. Repairing these DSBs via a homologous recombination (HR) mechanism primarily leads to gene conversion rather than to GCRs.^[^ ^25^ ^]^ However, the prevalent utilization of microhomology at both the initiation and target sites of translocations indicates the involvement of A‐EJ in mediating nontargeted translocations. The cleavage of FBs by SbcCD typically generates DSBs with sticky ends, which are favorable for A‐EJ repair.^[^ ^39^ ^]^ Furthermore, our previous study revealed that the competition between HR and A‐EJ depends on the complexity of DSBs, with A‐EJ being more proficient in repairing highly complex DSBs.^[^ ^43^ ^]^ It is reasonable to assume that the co‐occurrence of FB‐derived and Cas9‐induced DSBs further increases the complexity of DSB loci, facilitating the utilization of A‐EJ. A‐EJ has been widely recognized as a major contributor to chromosomal translocations.^[^ ^44^, ^45^ ^]^ Therefore, the intramolecular SSA combined with A‐EJ serves as the primary source for nontargeted translocations. Notably, while A‐EJ typically utilizes microhomologous sequences with lengths of less than 20 bp, we observed that ≈7% of the homologous sequences exceeded this length limit (Figure 2D). Considering SSA's ability to utilize longer homologies, it is plausible that SSA and/or other HR mechanisms also contribute to DSB repair but to a lesser extent.

Notably, the introduction of homologues of IR loci effectively mitigated nontargeted translocations by reducing the formation of FBs (Figure 3B–D; Figure S5, Supporting Information). Among these homologues, l1–4 may affect the palindromic pairing of *lacI*‐IR, which is supported by the limited mitigating effect of l5, a spacer homologue (Figure 3B). The binding of these homologues to IRs might impede or alter FB formation in a manner that prevents recognition by SbcCD. In contrast, k1–4 homologues are homologous to sequences located between Cas9 targets and IRs; hence, they are more likely to disrupt the extension of end resection from Cas9 targets to IRs. It is worth noting that both inter‐ and intramolecular SSA rely on end resection. Thus, it is plausible that homologues positioned between Cas9 targets and IRs might also possess the ability to mitigate GCRs mediated by intermolecular SSA.

Our results demonstrate that Cas9 cleavage near IRs can significantly increase the frequency of genome‐wide mutations, particularly SVs (Figure 4B,C). Two potential explanations for this phenomenon are proposed: one involves off‐target effects, whereas the other suggests that Cas9 cleavage enhances the overall activity of A‐EJ in cells, thereby rendering other naturally occurring DSBs more susceptible to repair by A‐EJ. Notably, the introduction of homologue l4 effectively inhibited the occurrence of genome‐wide SVs (Figure 4B,C). Given that l4 does not affect Cas9 cleavage, off‐target effects are not responsible for genome‐wide SVs. Therefore, l4 likely reduces the overall activity of A‐EJ by decreasing the level of FB‐derived DSBs. Furthermore, when subjected to both Cas9 cleavage and l4, the reporter strains presented rates of SNP and InDel mutations comparable to those observed in control strains (4.4 vs 3.8 mutations/genome), even exhibiting a slight decrease compared to strains subjected to Cas9 cleavage alone (4.4 vs 5.8 mutations/genome) (Figure 4C). These findings suggest that introducing homologues not only prevents genome‐wide SVs, but also avoids additional genome‐wide mutations. Thus, introducing homologues of IR loci may represent a viable strategy for mitigating GCRs during CRISPR‐mediated genome editing. However, GCRs could result from unintended off‐target effects of CRISPR‐mediated genome editing. This strategy might be less suitable in this context owing to the lack of definitive IR loci for designing homologues.

The efficiency of CRISPR‐Cas9‐mediated gene knock‐in and knockout remains largely unaffected by the introduction of homologues (Figure 4A). Gene editing via knock‐in and knockout relies primarily on the HR mechanism, where end resection plays a crucial role in recruiting HR‐related proteins to DSB sites. Exo‐mediated long‐range end resection is not essential for HR; ^42^ therefore, knock‐in and knockout events might predominantly depend on local resection at Cas9 target sites. Given that homologues of IR loci mainly interfere with end resection far from Cas9 target sites, their impact on CRISPR‐Cas9‐mediated genome editing is reasonably minimal. On the other hand, donor templates used in CRISPR‐Cas9‐mediated precise genome editing resulted in only a limited reduction in translocations (Figure 3E), significantly less effective than homologues of IR loci (Figure 3B). This phenomenon could be attributed to the fact that end resection occurs prior to HR and proceeds at a relatively slow pace (≈4 kb h^−1^).^[^ ^46^, ^47^ ^]^ When the HR process at Cas9 target sites has been successfully completed, end resection might still be ongoing toward IRs, which could explain the persistence of translocations during CRISPR‐Cas9‐mediated precise genome editing. This explanation is further supported by the observation that FB formation was not significantly reduced in the presence of the donor template dm6 (Figure 3D).

In summary, we have successfully developed a method for the detection of nontargeted translocations, revealed an additional genetic risk associated with CRISPR‐Cas9‐meiated editing and its underlying mechanism, and devised an innovative approach for mitigating this risk. These fundamental discoveries provide novel insights into the genetic risk linked to CRISPR gene editing and suggest a viable strategy for developing genetically safer CRISPR systems.

## 4 Experimental Section

### Bacterial Strains and Culture Conditions

The bacterial strains used in this study were derived from *E.coli* K12 MG1655 and cultured at either 37°C or 30°C in Luria–Bertani (LB) medium supplemented with apramycin (50 µg mL^−1^; cat no: A600090, Sangon Biotech, Shanghai, China) and chloramphenicol (50 µg mL^−1^; cat no: A100230, Sangon Biotech) when necessary.^[^ ^48^ ^]^

### Construction of Reporter Systems for Nontargeted Translocations

The PCR primers used, listed in Table S2 (Supporting Information), were designed utilizing the web‐based software J5 Device Editor available at https://j5.jbei.org/. The DNA segments were amplified through a PCR instrument (T100 Thermal Cycler, Bio‐Rad, Hercules, USA) with KOD‐Plus‐Neo polymerase (cat no: KOD‐401, Takara, Osaka, Japan). The restriction endonuclease BsaI (cat no: ER0291) and T4 ligase (cat no: 15224025), both procured from Thermo‐Fisher Scientific (Waltham, MA, USA), were used. All the plasmids utilized in this study were constructed using the Golden Gate method,^[^ ^49^ ^]^ as specified in Table S3 (Supporting Information).

For the construction of the basic reporter system, the sequences were employed within the *lacZ* and *mhpR* genes as N20 sequences of guide RNA (gRNA) and the *amp* and *lacI::kan* genes as the knock‐in sequences in plasmid‐encoded single guide RNAs (psgRNA), generating the plasgRNA and pmlksgRNA plasmids, respectively. Initially, strain MG1655 was transfected with plasgRNA and pRedCas9, followed by activation of the CRISPR‐Cas9 system at 30°C through the addition of 2 g L^−1^ arabinose. After screening for the knock‐in strain via PCR amplification and DNA sequencing, multiplegeneration culture at 37°C was employed to eliminate these plasmids. This resulting strain was further transfected with both pmlksgRNA and pRedCas9. Following the same procedure, a transgenic strain harboring the *amp:lacO:lacI:spacer:lacI:lacO:kan* was successfully established. Similarly, we introduced mutations of the *sbcD*, *recA*, and *recB* genes into the basic reporter strain using prsgRNA, with the sequences within these genes being N20 sequences. The short‐IR reporter system is generally constructed according to the protocol for the basic reporter system. However, the right *lacI* gene and 405‐bp spacer were replaced with various lengths of the 5’‐region of the *lacI* gene and spacers, including 400/50 bp, 200/50 bp, 100/50 bp, and 20/0 bp *lacI* genes/spacers. To construct a genome‐wide reporter system, we first knocked out the intrinsic *lacI* and *lacO* genes in the genome, and then introduced a construct consisting of *lacI::lacI* (20 bp)::*lacO*::*kan* into the genome alongside with sgRNA plasmids designed for 113 genome‐wide loci (Table S2, Supporting Information).

### Dual Screening for Ampicillin (Amp) and Kanamycin (Kan) Resistance

The CRISPR‐Cas9 system was activated by culturing the reporter strains in liquid medium supplemented with 2 g L^−1^ arabinose at 30°C for 3 h. The strains in which the CRISPR‐Cas9 system without N20 sequences was induced by arabinose were used as a negative control. The strains were subsequently diluted and plated on control LB agar plates as well as selection plates containing 100 µg mL^−1^ Amp (cat no: A610028, Sangon Biotech) and 50 µg mL^−1^ Kan (cat no: A600286, Sangon Biotech), followed by overnight incubation at 37 °C. Colony‐forming units were manually counted using a dissecting microscope (ES‐18BZL, Motic, Xiamen, China). Subsequently, the Amp‐ and Kan‐resistant clones were validated through reculturing in LB liquid medium supplemented with the same concentrations of Amp and Kan. The mutation rate was determined by dividing the number of positive clones on the Amp and Kan selection plates by that on the control LB plates. The final data represent the average from five independent experiments.

### Detection of Mutation Types

The types of *lacI*‐IR mutations were identified using PCR amplification and Sanger sequencing. The employed PCR primers used are listed in Table S4 (Supporting Information). B‐type mutations in Amp‐ and Kan‐resistant clones were initially determined on basis of spacer retention, followed by verification through sequencing of the *lacI* genes. In the absence of the spacer, we first examined the retention of the *amp* and *kan* genes at their original loci via PCR amplification using forward primers specific to the *amp* or *kan* genes paired with a set of reverse primers targeting their respective flanking genome sequences combined with a matrix of primer pairs positioned in a tiled manner (Table S4, Supporting Information). Through pilot experiments, it was observed that the absence of amplicons of both the *kan* gene and its flanking genome sequence of 3 kb can indicate a translocation event at the *kan* locus (C‐type). Conversely, the presence of an amplicon signifies an A‐type mutation. This finding was further validated via PCR amplification using forward primers at the *amp* or *kan* genes paired with a set of reverse primers at the *lacI* gene. In A‐type mutations, some junctions between two residual *lacI* genes can be identified via PCR amplification using primers at *amp*/*kan* genes paired with those at the extra part of one *lacI* gene relative to the other.

### Characterization of Microhomology at Translocation Target and Initiation Sites

Thermal asymmetric interlaced polymerase chain reaction (tail‐PCR) was employed to amplify the junctions at the translocation initiation and target sites. The PCR primers utilized in this study are listed in Table S4 (Supporting Information). The resulting amplicons were subjected to sequencing analysis by Sangon Bioteck, and identification of the junction sequences at translocation initiation and target sites was achieved through alignment with the *E. coli* genome via the National Center for Biotechnology Information (NCBI) alignment tool. Molecular characterization of these junction sequences was performed using DNAMAN sequence analysis software (https://www.Lynnon.com/dnaman.html).

### Identification of Foldback Structures

Structural bisulfate assays have been widely utilized for the detection of non‐B DNA structures, including hairpin, foldback, and cruciform configurations. These structures comprise single‐stranded loops, wherein cytosine residues can be specifically converted to uracil via bisulfite treatment. The resulting uracil can then be assessed through DNA sequencing.^[^ ^50^ ^]^ In this study, the identification of foldback structures was conducted according to a previously published method.^[^ ^51^ ^]^ Genomic DNA was extracted from 4 mL of bacterial culture (OD = 0.6) using the Rapid Bacterial Genomic DNA Isolation Kit (cat no: B518225, Sangon Biotech) following the manufacturer's instructions. Subsequently, 90 µL of genomic DNA was incubated with 12.5 µL of 20 mM hydroquinone and 397.5 µL of 4 M sodium bisulfite (pH 5.5) in opaque tubes for 36 h at 55°C while preventing evaporation with mineral oil. An EZ‐10 Column DNA Purification Kit (cat no: B610367, Sangon Biotech) was used to recover the bisulfite‐converted DNA. The spacers within IRs were PCR amplified, and the resulting amplicons were purified using the EZ‐10 Column DNA Purification Kit and then cloned and inserted into the pEASY‐Blunt Cloning Vector (cat no: CB101, TransGen Biotech, Beijing, China). After being transfected into DH5a cells, 48 positive colonies were randomly selected for sequencing analysis. The occurrence of cytosine conversion within spacer regions was determined using NCBI.

### Whole‐Genome Sequencing of *Escherichia Coli*

Whole‐genome sequencing was conducted by Biomarker Technologies Company (Beijing, China) using Oxford Nanopore technology. The filtered reads were assembled using Canu software (version 1.5), after which the assembly of the genome was cyclized via Circulator (version 1.5.5). The GenBlastA program (version 1.0.4) was used to scan the entire genome for the identification of genetic alterations.

### Assessment of CRISPR‐Cas9 Editing Efficiency

The donor templates used for gene knock‐in and knockout at distances of 2, 3.5, and 6 kb are listed in Table S2 (Supporting Information). The CRISPR‐Cas9 system, with or without homologues l4 and k4, was activated using the same procedure mentioned above but for a duration of 24 h. Subsequently, the bacteria were transferred onto clone culture medium to detect knock‐in and knockout events using PCR analysis. The final data represent the average results obtained from three independent experiments, with each experiment consisting of 48 clones.

### Statistical Analysis

The mean ± standard deviation was used to report the data for the rates of mutations and translocations. The normality of the data was determined using the Kolmogorov–Smirnov test. Student's *t*‐test or the Mann–Whitney U test (SPSS, version 23.0, IBM, New York USA) was used to assess differences between two groups for normally distributed or nonnormally distributed data, respectively. Significance was considered at *P* < 0.05. The correlation between two groups of data was assessed by the Pearson correlation coefficient.

## Conflict of Interest

The authors declare no conflict of interest.

## Author Contributions

Z.H. and Q.Y. contributed equally to this work. P.B., T.W., L.W. designed the study; Z.H. and P.B. designed IR reporter systems; T.W. and Z.H. designed non‐IR reporter systems; Z.H., Q.Y., and M.W. performed the experiments; T.W., Z.H., and Q.Y. analyzed the data and made the charts; F.L. provided crucial suggestions on DSB repair; P.B. wrote the paper.

## Supporting information



Supporting Information

Supplemental Table 1

Supplemental Table 2

Supplemental Table 3

Supplemental Table 4

## Data Availability

The data that support the findings of this study are available in the supplementary material of this article.
