# Supplementary material for: Assessment and Mitigation of CRISPR‐Cas9‐Induced Nontargeted Translocations
Source: Adv Sci (Weinh). 2025 Apr 11;12(21):2414415. doi: 10.1002/advs.202414415 (PMC12140377; doi:10.1002/advs.202414415)
Supplement: Supplementary file 1 — Supporting Information [file ADVS-12-2414415-s005.docx]

**

**

**Figure S1. Schematic representation of inverted repeat-mediated GCRs.** DSB, DNA double-strand break; SSA, single-strand annealing; ID, inverted dicentric chromosome; FB, foldback structure; MRX/Sea2, mre11-Rad50-Xrs2/Sae2 complex; GCRs, gross chromosomal rearrangements.

**
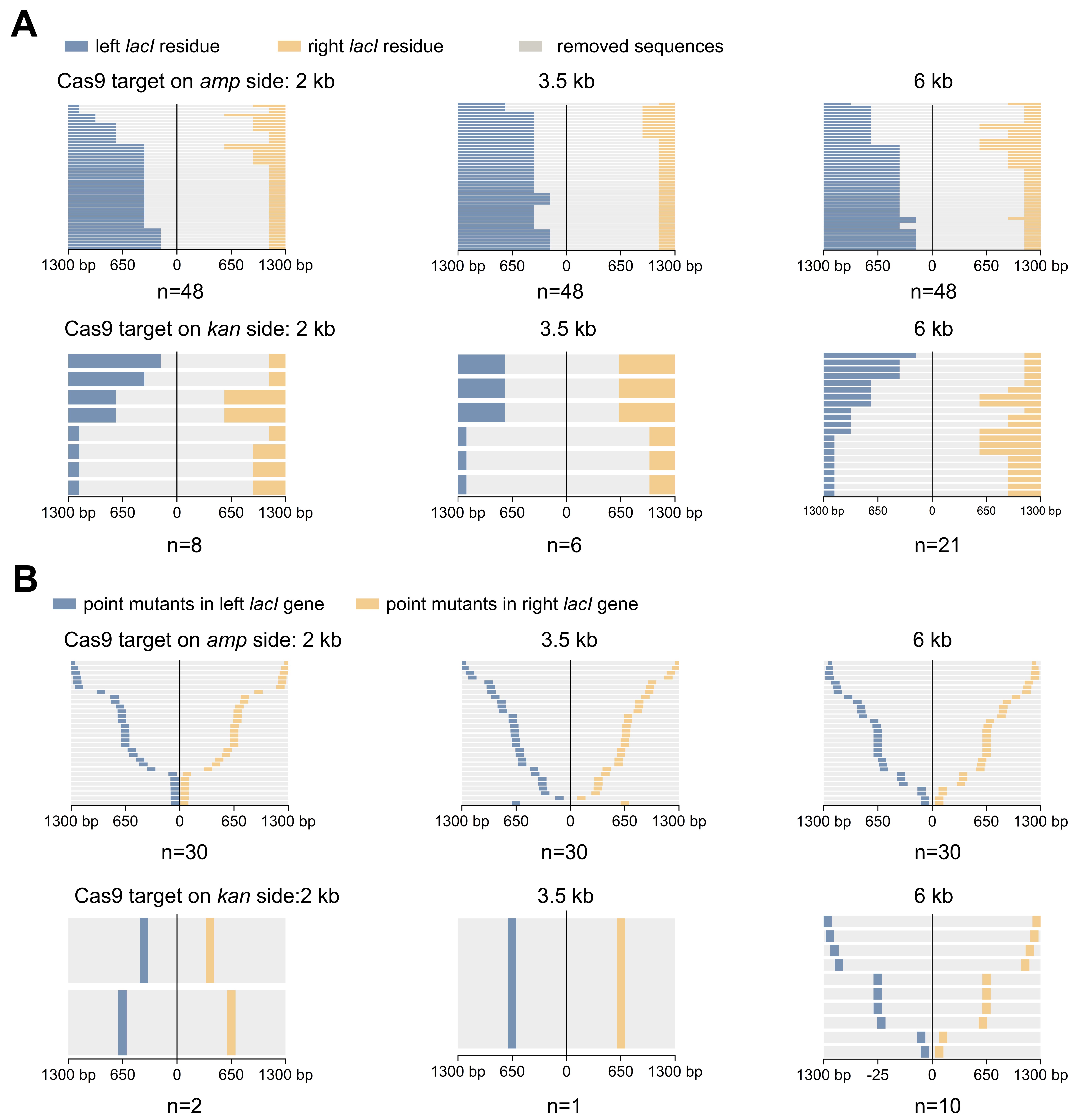
**

**Figure S2. Sequence characteristics of A- and B-type mutations.**

(A) Segment deletions spanning two *lacI* genes (A-type) when Cas9 cleavage occurs at the indicated distances from the IR. (B) Distribution of double point mutations in two *lacI* genes (B-type) when Cas9 cleavage occurs at the indicated distances.


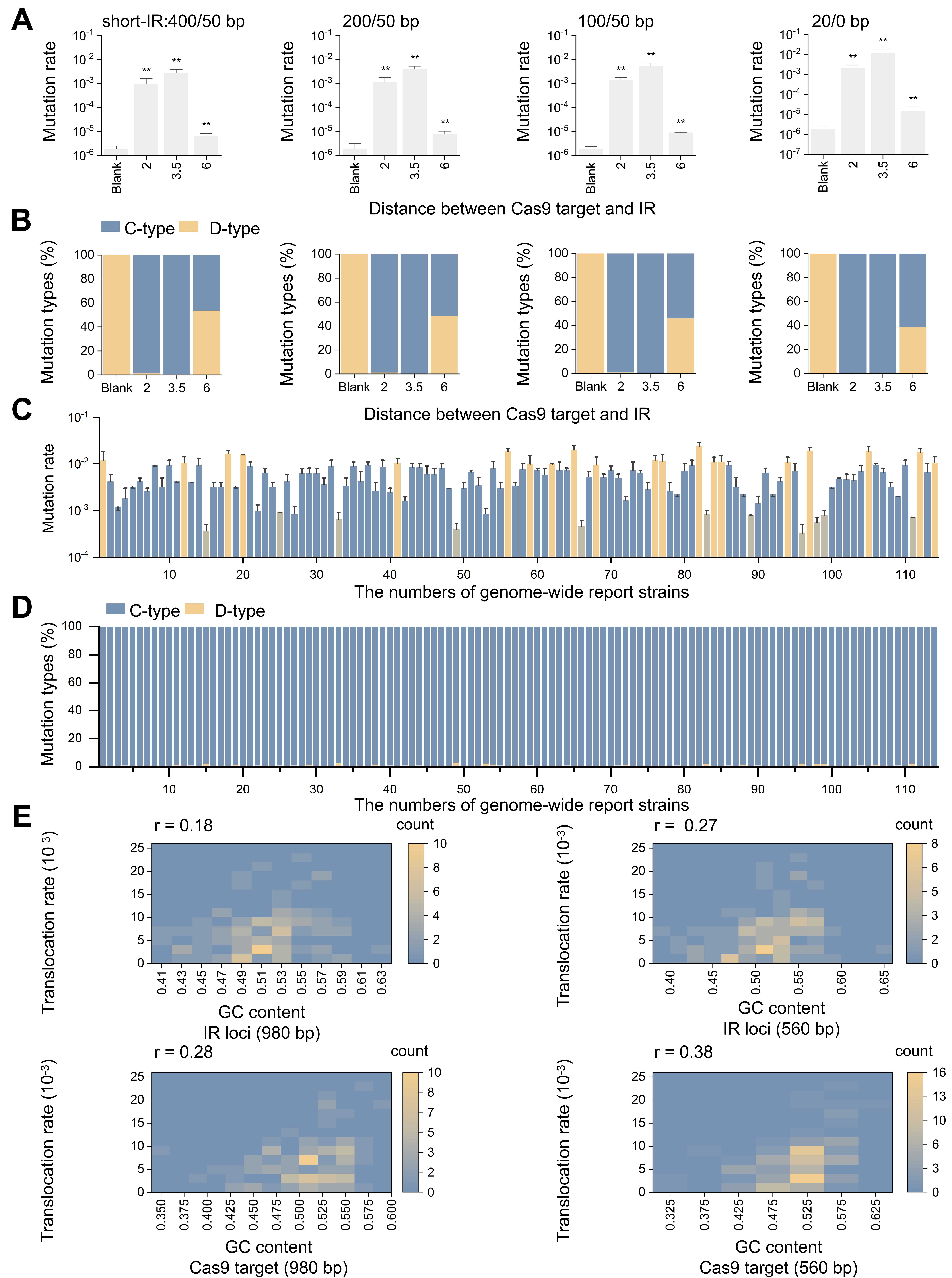


**Figure S3. Mutations induced by Cas9 cleavage near IRs in short-IR and genome-wide reporter systems.** Rates of Kan- and Amp-resistant clones (A) and proportions of C- and D-type mutations (B) in short-IR reporter strains subjected to Cas9 cleavage at the indicated distances (*n* = 5, **, *P* < 0.01). (C) Rates of Kan-resistant clones in genome-wide reporter strains subjected to Cas9 cleavage at a distance of approximately 3 kb from IRs (*n* = 5, *P* < 0.01 compared to their respective Blank controls). (D) Proportions of C- and D-type mutations in genome-wide reporter strains subjected to Cas9 cleavage (*n* = 5). (E) Heatmaps indicating translocation rates associated with the GC content at Cas9 target sites and IR loci in genome-wide reporter system. Black, control strains without CRISPR-Cas9 system.

**
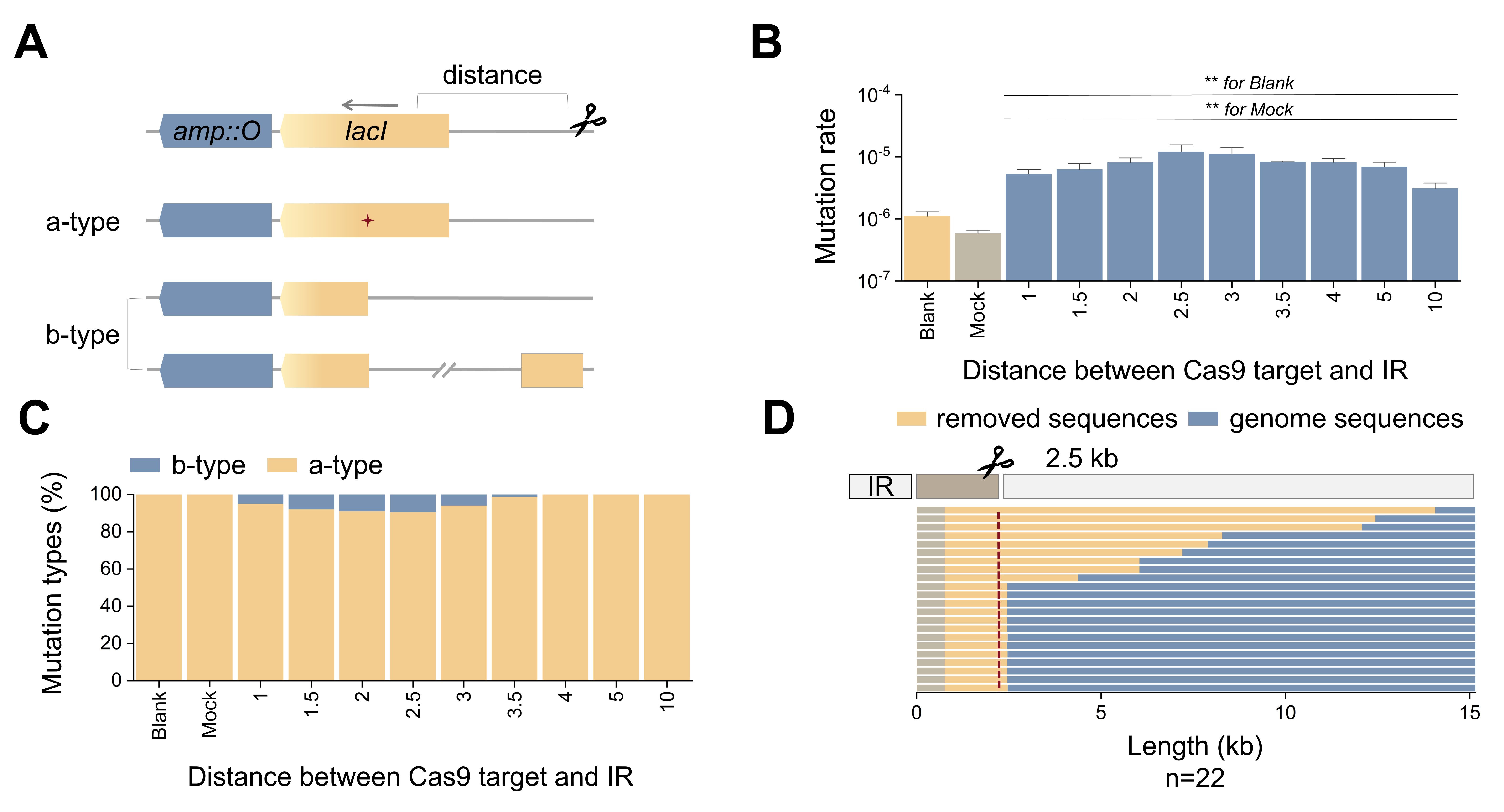
**

**Figure S4. CRISPR-Cas9-mediated deletions/translocations in a non-IR reporter system.**

(A) Schematic representation of the non-IR reporter system, where b-type mutations consist of partial deletions of the *lacI* gene and translocations at a ratio of 22:9. (B) Rates of Amp-resistant clones induced by Cas9 cleavage (*n* = 5, **, *p* < 0.01). (C) Proportions of a- and b-type mutations induced by Cas9 cleavage at the indicated distances (*n* = 5). (D) Lengths of removed sequences at the *lacI* locus in b-type mutations induced by Cas9 cleavage at a distance of 2.5 kb. Black, control strains without CRISPR-Cas9 system. Mock, control strains with CRISPR-Cas9 system lacking N20 guide sequences.

**
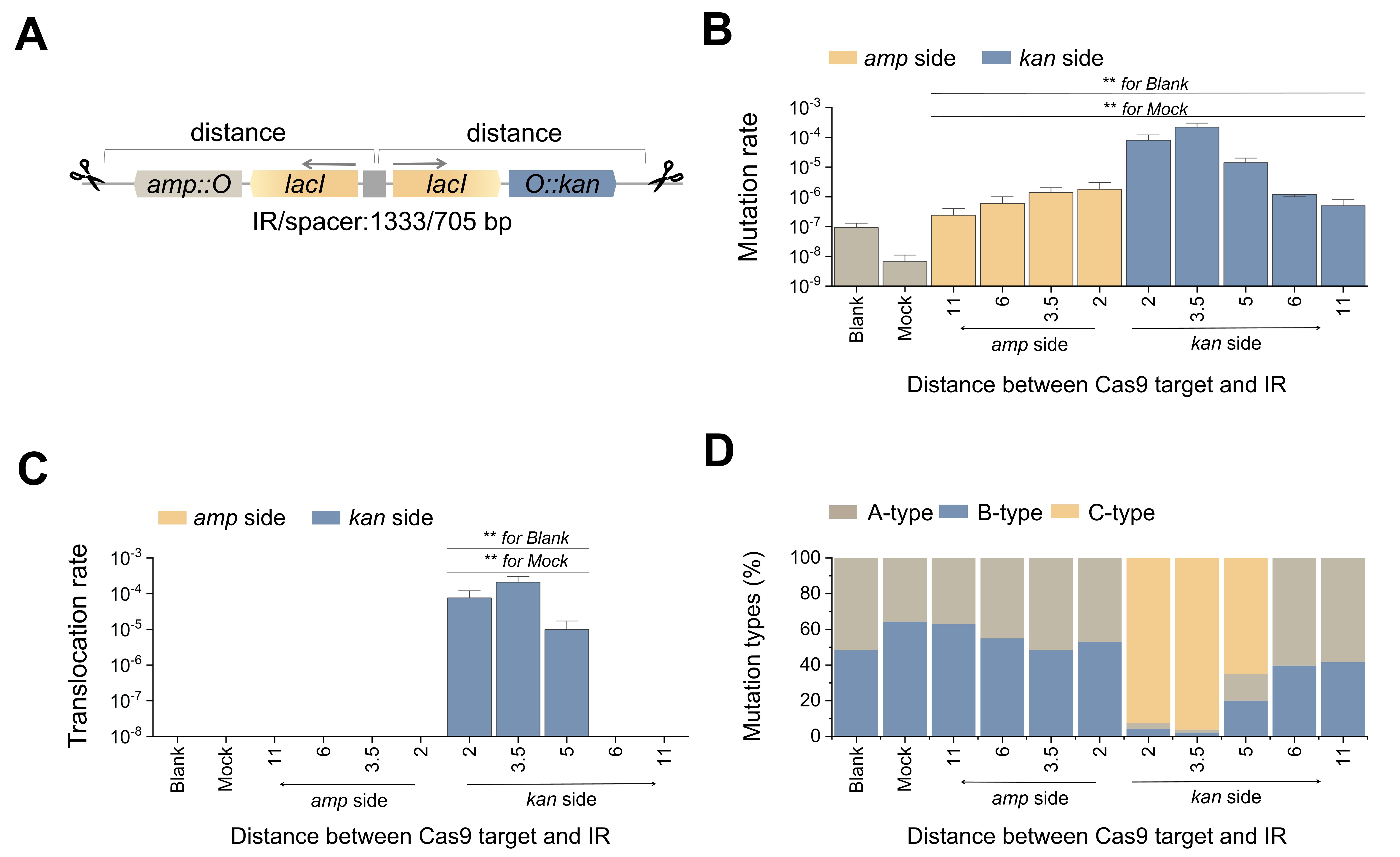
**

**Figure S5. CRISPR-Cas9-induced translocations in an IR reporter system with a 705-bp spacer.**

(A) Schematic representation of the *lacI*-IR reporter system with a 705-bp spacer. (B) Rates of Amp- and Kan- resistant clones induced by Cas9 cleavage at the indicated distances (*n* = 5, **, *P* < 0.01). (C) Translocation rates induced by Cas9 cleavage at the indicated distances (*n* = 5, **, *P* < 0.01). (D) Proportions of A-, B-, and C-type mutations induced by Cas9 cleavage at the indicated distances (*n* = 5). Black, control strains without CRISPR-Cas9 system. Mock, control strains with CRISPR-Cas9 system lacking N20 guide sequences.





**Figure S6. Mitigation of translocations by homologues of IR loci** **in short-IR and genome-wide reporter systems.**

Translocation rates in four short-IR reporter strains in the presence of their respective homologues of IRs (A) or homologue k4 (B). Translocation rates in ten randomly selected genome-wide reporter strains in the presence of IR homologue (40 bp) (C) or homologues of IR-flanked sequences (lengths ranged from 96 bp to 126 bp) (D). Blank, control strains without homologues.


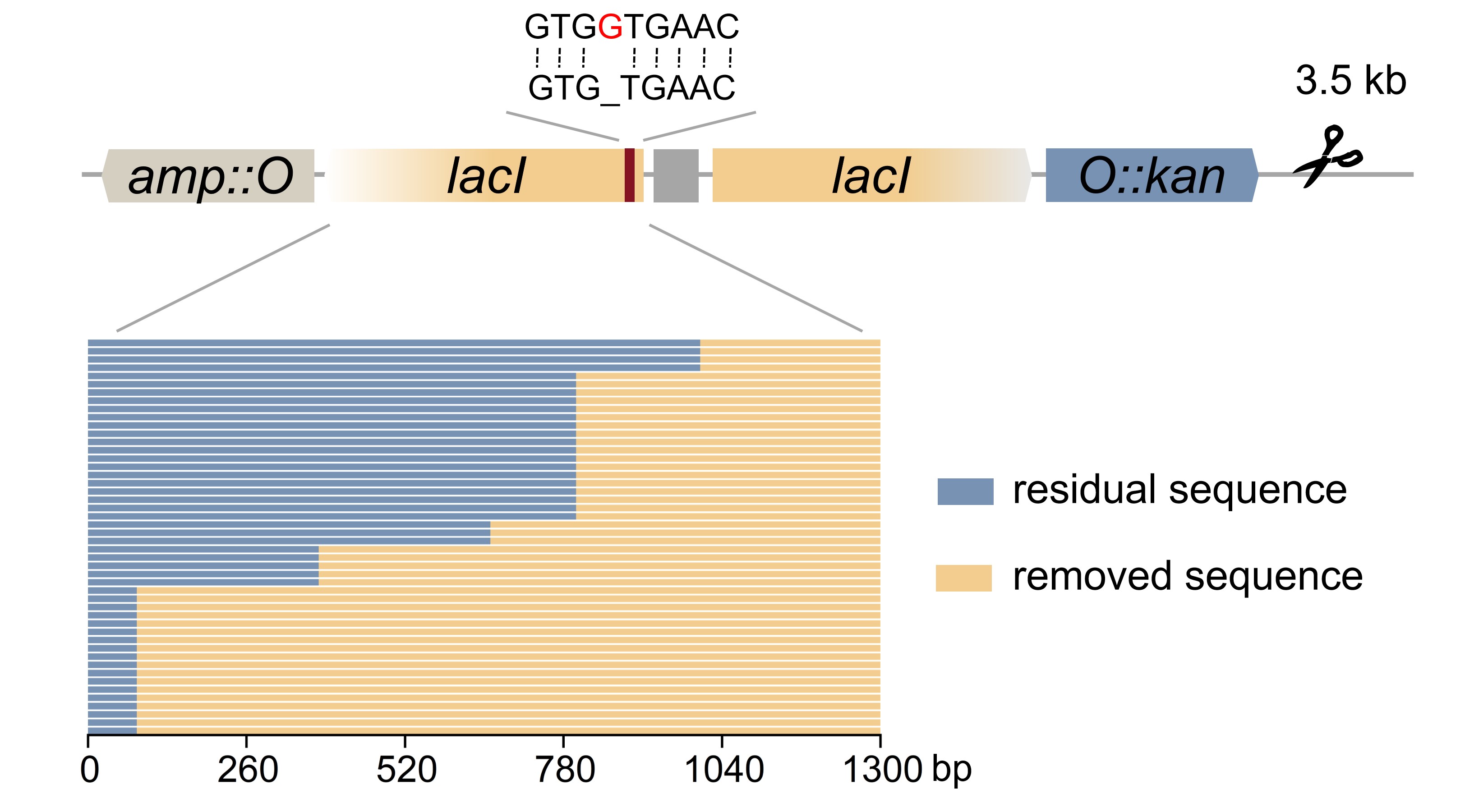


**Figure S7. The basic IR reporter system with a single-base frameshift mutation in the left *lacI* gene** **(98 bp downstream of the ATG) and residual lengths of the mutated gene in the C-type mutations.**
